# Thymol Inhibits Biofilm Formation, Eliminates Pre-Existing Biofilms, and Enhances Clearance of Methicillin-Resistant *Staphylococcus aureus* (MRSA) in a Mouse Peritoneal Implant Infection Model

**DOI:** 10.3390/microorganisms8010099

**Published:** 2020-01-10

**Authors:** Zhongwei Yuan, Yuyun Dai, Ping Ouyang, Tayyab Rehman, Sajjad Hussain, Tianyi Zhang, Zhongqiong Yin, Hualin Fu, Juchun Lin, Changliang He, Cheng Lv, Xiaoxia Liang, Gang Shu, Xu Song, Lixia Li, Yuanfeng Zou, Lizi Yin

**Affiliations:** College of Veterinary Medicine, Sichuan Agriculture University, Huimin Lu 211, Wenjiang 611130, China; yuanzhongwei_sicau@163.com (Z.Y.); dyy15928065710@163.com (Y.D.); Ouyang.ping@live.cn (P.O.); miantyb30@gmail.com (T.R.); sajjadsheikh936@gmail.com (S.H.); 0829zty@gmail.com (T.Z.); yinzhongq@163.com (Z.Y.); juchunlin@126.com (J.L.); lorri190@126.com (C.H.); Lvcheng1980@163.com (C.L.); liangxiaoxia@sicau.edu.cn (X.L.); cndyx2005@163.com (G.S.); songx@sicau.edu.cn (X.S.); lilixia905@163.com (L.L.); yuanfengzou860315@163.com (Y.Z.)

**Keywords:** MRSA, PIA, eDNA, biofilm, thymol, vancomycin, antibiotic resistance

## Abstract

Methicillin-resistant *Staphylococcus aureus* (MRSA) is a common human pathogen that causes several difficult-to-treat infections, including biofilm-associated infections. The biofilm-forming ability of *S. aureus* plays a pivotal role in its resistance to most currently available antibiotics, including vancomycin, which is the first-choice drug for treating MRSA infections. In this study, the ability of thymol (a monoterpenoid phenol isolated from plants) to inhibit biofilm formation and to eliminate mature biofilms, was assessed. We found that thymol could inhibit biofilm formation and remove mature biofilms by inhibiting the production of polysaccharide intracellular adhesin (PIA) and the release of extracellular DNA (eDNA). However, cotreatment with thymol and vancomycin was more effective at eliminating MRSA biofilms, in a mouse infection model, than monotherapy with vancomycin. Comparative histopathological analyses revealed that thymol reduced the pathological changes and inflammatory responses in the wounds. Assessments of white blood cell counts and serum TNF-α and IL-6 levels showed reduced inflammation and an increased immune response following treatment with thymol and vancomycin. These results indicate that combinatorial treatment with thymol and vancomycin has the potential to serve as a more effective therapy for MRSA biofilm-associated infections than vancomycin monotherapy.

## 1. Introduction

*Staphylococcus aureus* is an opportunistic, gram-positive bacterium that is a major cause of nosocomial infections [1]. *S. aureus* can cause several different infections in humans and animals, ranging from pneumonia to sepsis and endocarditis [2]. *S. aureus* infections are treated with various antibiotics. However, the indiscriminate use of antibiotics has resulted in the emergence of antibiotic-resistant strains, including methicillin-resistant *S. aureus* (MRSA), which is a serious public health hazard [3]. Patients with community-acquired and nosocomial infections are frequent carriers of MRSA [4].

The proportion of MRSA-attributable, hospital-acquired bacterial infections has been increasing every year, and MRSA is a serious threat to postoperative patients [5]. Most patients with postoperative MRSA infections die due to the infection, rather than due to surgical complications, as the fatality rate of MRSA infections is high [6]. Approximately 39–51% of the pathogens causing infections at surgical sites have been estimated as being resistant to conventional antibiotics in the United States [7,8].

Previous studies have shown that MRSA can form biofilms on infected tissues and medical instruments, thus it is gradually becoming even more difficult to treat MRSA infections [9,10,11]. Biofilms are formed by aggregates of bacteria that adhere to various surfaces; they are mainly composed of DNA, proteins and extracellular polysaccharides [12,13]. Biofilm formation is primarily initiated by the adherence of planktonic cells [14]. After biofilm maturation, some bacterial cells disperse, and these dormant cells are reconverted to planktonic cells [15]. Compared with planktonic cells, biofilm-embedded, bacterial cells exhibit lower growth rates, more frequent cellular communication, and lower sensitivities to antibiotics, which makes them more difficult to eradicate [12,16]. Bacteria in a biofilm are more resistant to antibiotics (up to 1000-fold), and can evade the immune system of the host. Thus, biofilm-associated infections can be persistent [17] and even fatal [10]. Previous studies have demonstrated that *S. aureus* biofilms show a reduced permeability to vancomycin [18]. Compared to typical bacterial infections, biofilm-associated infections are much more laborious to treat [19]. Given the increasing incidence of multidrug resistance and our current knowledge of the tenacious nature of biofilms, it is necessary to develop novel strategies and drugs that target biofilm-associated MRSA infections.

Plant-derived compounds have been widely used to combat microbial infections because they are inexpensive and easy to extract [20,21]. The traditional Chinese medicine, 2-isopropyl-5-methylphenol (IPMP), commonly known as thymol, is a monoterpene phenol isolated from plants. It is a component of the essential oils extracted from various plants in the *Lamiaceae*, *Verbenaceae*, and *Scrophulariaceae* families, and it exhibits anti-inflammatory, antioxidant and potent antimicrobial properties [22,23,24]. In our previous studies [25] we showed the potent effects of thymol against MRSA infections. Thymol inhibits bacterial growth by altering the membrane permeability and disturbing both protein synthesis and binary fission. At subinhibitory concentrations, thymol also reduces biofilm formation [25]. Therefore, in this study, we first investigated the ability of thymol to inhibit biofilm formation and to eliminate mature biofilms in vitro. Second, we evaluated the effects of thymol on extracellular DNA (eDNA) release, polysaccharide intercellular adhesin (PIA) production, and the expression of biofilm-associated genes. Finally, but most importantly, we evaluated a combination of thymol and vancomycin for the treatment of MRSA biofilm infections in a mouse model.

## 2. Results

### 2.1. Effects of Thymol on Growth and the Results of Thymol-Vancomycin Checkerboard Assay

**Figure 1:**
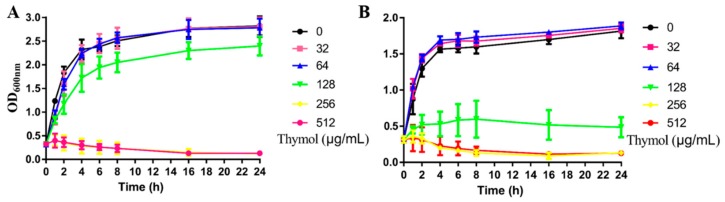
Growth curves of TCH1516 in brain heart infusion (BHI) containing different concentrations of thymol. (**A**) Under shaking conditions. (**B**) Under static conditions.

A growth curve (Figure 1) showed that thymol at a concentration of 0, 8, 16, 32 and 64 μg/mL did not directly inhibit the growth of the MRSA strain, TCH1516. However, at a concentration of 128 μg/mL, thymol slightly inhibited the growth of TCH1516, and at concentrations of 256 and 512 μg/mL, it completely inhibited the growth of this strain. Based on the results of the checkerboard assay, the fractional inhibitory concentration index (FICI) was calculated as 1.0 (128/256 + 1/2 = 1.0), indicating that the combination of thymol and vancomycin hydrochloride showed an additive effect (Table S1).

### 2.2. Thymol Inhibited Biofilm Formation by Strain TCH1516

**Figure 2:**
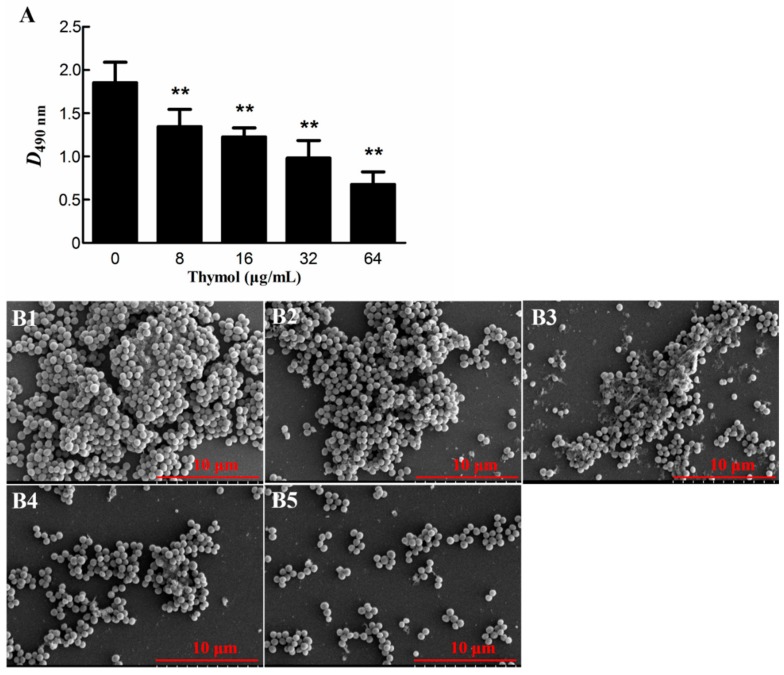
Inhibitory effect of thymol on biofilm formation by TCH1516. (**A**) Results of crystal violet staining. (**B**) Scanning electron microscopy (SEM) images. Biofilms formed by cells treated with 0, 8, 16, 32, and 64 μg/mL thymol are shown in B1, B2, B3, B4 and B5, respectively. ** *p* < 0.01 compared with the 0 μg/mL group.

The results of the biofilm experiment are shown in Figure 2A. The OD_490_ of the untreated group was much higher than that of the treated groups. Treatment with thymol at concentrations of 8 and 64 μg/mL decreased the TCH1516 biofilms by 27.0% ± 10.4% (*p* < 0.01) and 63.9% ± 5.1% (*p* < 0.01), respectively, when compared to the biofilms in the untreated group. Scanning electron microscopy (SEM) observations showed that the dish containing the 0 μg/mL group was densely covered by cells (Figure 2(B1)). However, as the thymol concentration increased, the number of cells decreased. In the group treated with 64 μg/mL thymol, only a small number of unaggregated cells was observed (Figure 2(B5)). These results showed that sub-MICs of thymol (8, 16, 32, and 64 μg/mL) significantly inhibited TCH1516 biofilm formation in a dose-dependent manner.

### 2.3. Thymol Disrupted Mature TCH1516 Biofilms 

**Figure 3:**
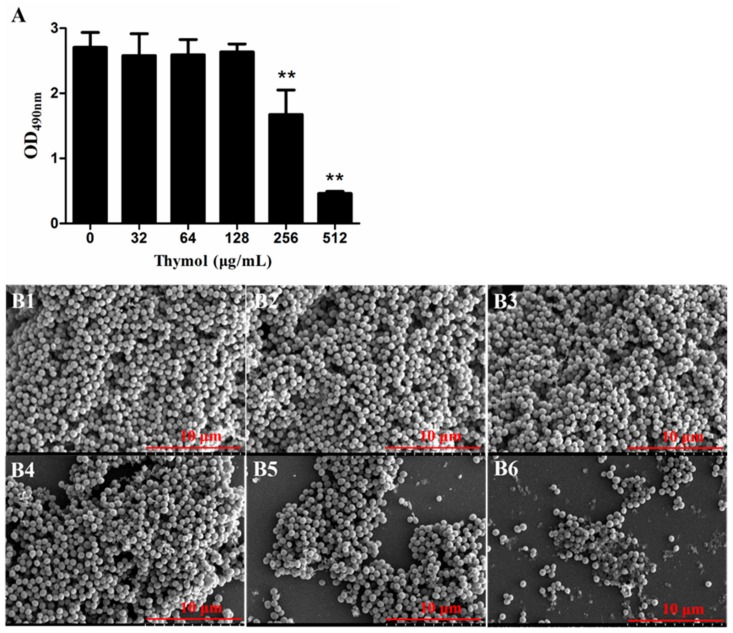
The biofilm removal effect of thymol on pre-existing TCH1516 biofilms. (**A**) Crystal violet staining of treated and untreated biofilms. (**B**) SEM images of treated and untreated biofilms. The biofilms in B1, B2, B3, B4, B5 and B6 were treated with 0, 32, 64, 128, 256 and 512 μg/mL thymol, respectively ** *p* < 0.01, compared with the untreated (0 μg/mL) biofilm.

A biofilm removal assay revealed that thymol, at concentrations of 256 and 512 μg/mL, effectively disrupted preformed biofilms. After treatment with sub-MICs (32, 64 and 128 μg/mL) of thymol for 24 h, the biofilms barely changed in comparison with the untreated biofilms (0 μg/mL). In contrast, biofilms treated with 256 and 512 μg/mL thymol decreased by 30.4% ± 7.6% and 82.9% ± 1.1%, respectively (Figure 3A). SEM showed an abundance of cells in the biofilms treated with 0, 32, 64 and 128 μg/mL thymol (Figure 3(B1–B4)), while the number of cells in the biofilms treated with 256 μg/mL was decreased, and a small amount of the PIA was exposed (Figure 3(B5)). In biofilms treated with 512 μg/mL thymol, the number of bacteria was significantly decreased, and many reticular PIAs were observed (Figure 3(B6)). As shown in Table S2, low concentrations of vancomycin hydrochloride promoted TCH1516 biofilm formation. However, when combined with thymol, vancomycin enhanced the biofilm removal effect.

### 2.4. PIA Production was Inhibited by Thymol

**Figure 4:**
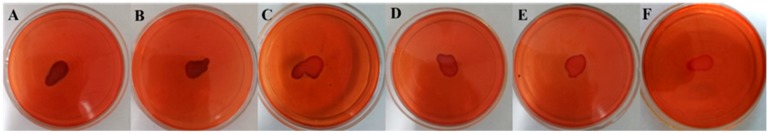
The effects of thymol on the production of polysaccharide intracellular adhesin (PIA) by TCH1516 biofilms. Plates shown in (**A**–**F**) were treated with 0, 8, 16, 32, 64 and 128 μg/mL thymol, respectively.

In a Congo red plate culture, PIA-positive bacterial colonies appeared black in color, while PIA-negative colonies appeared red in color. The results shown in Figure 4 reveal that as the thymol concentration increases, there is a gradual decrease in the synthesis of PIA, which indicates that thymol inhibits the synthesis of PIA in MRSA biofilms.

### 2.5. Thymol Decreased eDNA Release

**Figure 5:**
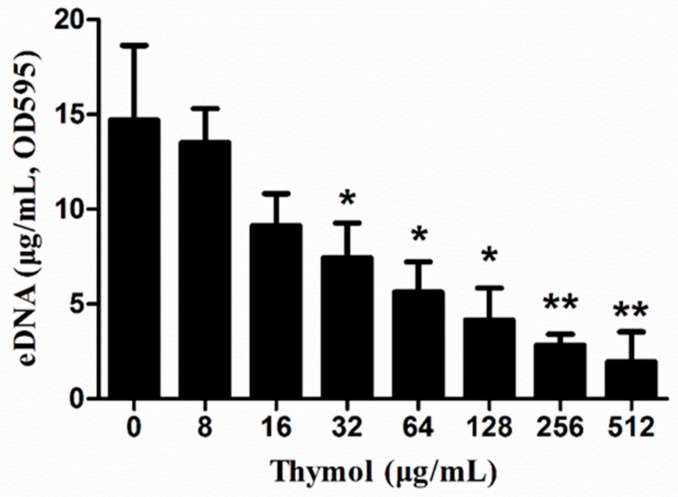
The effects of thymol on eDNA release by TCH1516 biofilms. * *p* < 0.05 or ** *p* < 0.01, compared with the 0 μg/mL group.

As shown in Figure 5, lower concentrations of thymol inhibited the release of eDNA from MRSA biofilms. This inhibitory effect was enhanced by an increase in thymol concentration. After treatment with 32 and 512 μg/mL thymol, eDNA release decreased by 49.4% ± 12.6% and 86.9% ± 10.9%, respectively, when compared to that in the 0 μg/mL group.

### 2.6. Thymol Inhibited the Transcription of Biofilm-Regulated Genes

**Figure 6:**
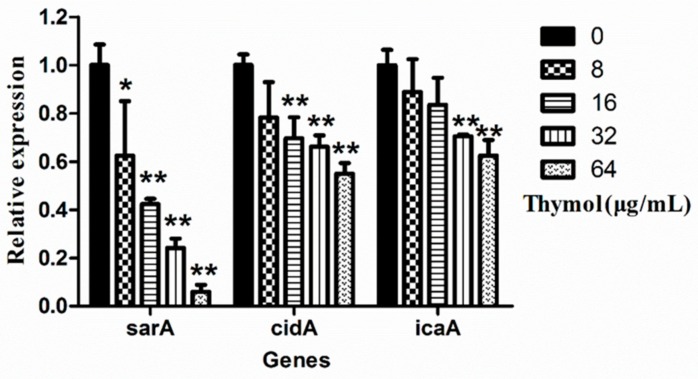
Expression of *ica*A, *cid*A and *sar*A in TCH1516 in the presence of various concentrations of thymol. ** *p* < 0.01 or * *p* < 0.05, compared with the same genes in the 0 μg/mL group.

The transcript levels of key biofilm-regulated genes (*sar*A, *cid*A and *ica*A), as well as the 16S rDNA gene in TCH1516 treated with various concentrations of thymol, were determined using a real-time polymerase chain reaction (RT-PCR), and are shown in Figure S1. The real-time PCR data showed a concentration-dependent relationship between thymol and the transcriptional levels of *sar*A, *cid*A and *ica*A (Figure 6). At 64 μg/mL, thymol significantly (*p* < 0.05) decreased the transcript levels of *sar*A, *cid*A and *ica*A by 94.0% ± 2.8% (*p* < 0.01), 45.0% ± 4.3% (*p* < 0.01), and 30.9% ± 17.8%, respectively, when compared with those in the untreated group.

### 2.7. Vancomycin Combined with Thymol Reduced Intraperitoneal Foreign-Body Biofilm Infection Caused by MRSA in Mice

#### 2.7.1. Vancomycin Combined with Thymol Reduced Bacterial Adhesion

**Figure 7:**
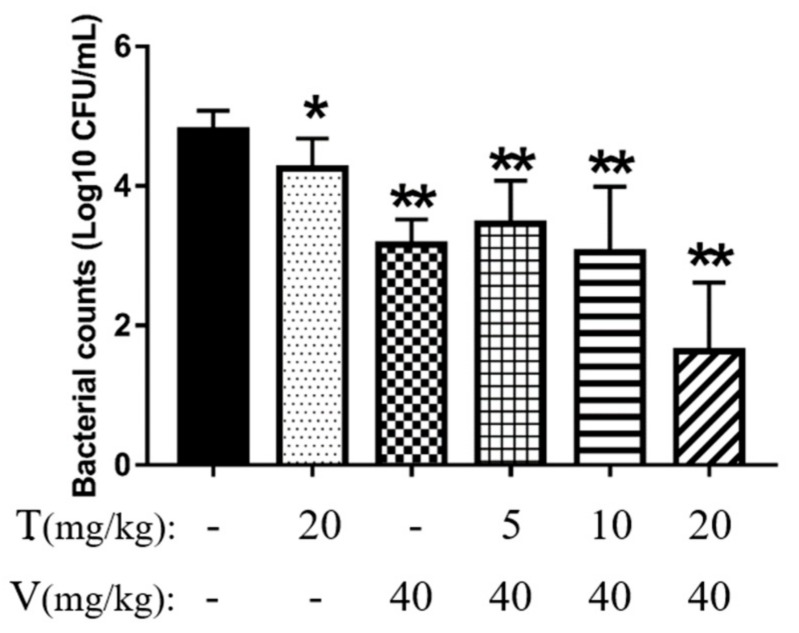
Bacterial counts in different mouse treatment groups. The results are expressed as the mean ± standard deviation (SD). * *p* < 0.05 and ** *p* < 0.01 indicate statistically significant and extremely significant differences compared with the PBS group. T, thymol; V, vancomycin hydrochloride.

First, the bacterial burden imposed by the biofilms on the implants in all six groups (PBS, thymol alone, vancomycin hydrochloride alone, low-dose combination treatment, middle-dose combination treatment and high-dose combination treatment groups) was evaluated after 24 h of treatment, by colony counting. The implants treated with a combination of thymol (20 mg/kg) and vancomycin (40 mg/kg) exhibited almost no bacterial adhesion (Figure 7), and the bacterial counts were significantly lower (by 99.7% ± 0.3%; *p* < 0.01) than those observed in the PBS group. Other treatments showed some effects, but were not as remarkable as those in the high-dose combination treatment group.

#### 2.7.2. Vancomycin in Combination with Thymol Eliminated Mature Biofilms

**Figure 8:**
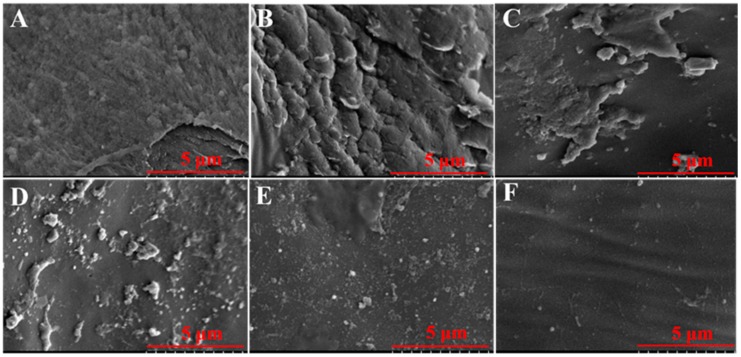
SEM images of peritoneal implants from mice treated with combinations of vancomycin and/or thymol. (**A**) PBS group, (**B**) thymol group (20 mg/kg), (**C**) vancomycin group (40 mg/kg), (**D**) low-dose combination treatment group (vancomycin hydrochloride 40 mg/kg + thymol 5 mg/kg). (**E**) middle-dose combination treatment group (vancomycin hydrochloride 40 mg/kg + thymol 10 mg/kg), and (**F**) high-dose combination treatment group (vancomycin hydrochloride 40 mg/kg + thymol 20 mg/kg).

Significant differences in biofilm clearance were also observed by SEM. In the PBS group, dense biofilms were observed, and the implant was completely covered with biofilm bacteria (Figure 8A). In the thymol-treated and vancomycin monotherapy groups, a thin layer of biofilm was still adhered to the implants (Figure 8B,C). In the high-dose combination treatment group, no biofilms were observed, and only a few scattered bacteria adhered (Figure 8F).

#### 2.7.3. Vancomycin in Combination with Thymol Relieved the Pathological Damage Caused by MRSA Infection

**Figure 9:**
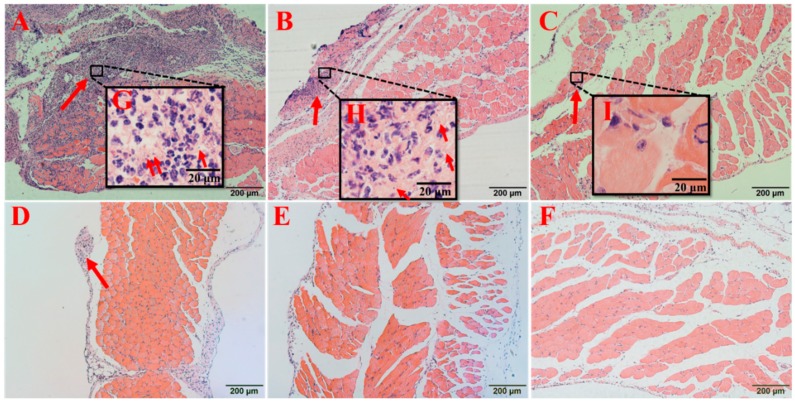
Pathological changes in the peritoneal tissues surrounding the implants after treatment. (**A**) PBS group, (**B**) thymol group (20 mg/kg), (**C**) vancomycin group (40 mg/kg), (**D**) low-dose combination treatment group (vancomycin hydrochloride 40 mg/kg + thymol 5 mg/kg), (**E**) middle-dose combination treatment group (vancomycin hydrochloride 40 mg/kg + thymol 10 mg/kg), and (**F**) high-dose combination treatment group (vancomycin hydrochloride 40 mg/kg + thymol 20 mg/kg). Magnification: 100× in (A–F); 1000× in (G–I). Red arrows express infiltration of inflammatory cells.

In the PBS group, the abdominal wall of the mouse was significantly thickened, the capillary walls were dilated, and the neutrophils and bacteria were observed between muscle cells (Figure 9A). In the thymol-treated group, the structure of the muscle was more intact, although some inflammatory cells and bacteria were present (Figure 9B). In the vancomycin-treated group, a few inflammatory cells were observed, but no bacteria were detected (Figure 9C). In all three combination treatment groups, there were fewer inflammatory cells compared to that in the PBS group, especially in the high-dose combination group, in which only a few inflammatory cells were observed (Figure 9F). Bacteria were isolated from the tissues in the PBS and thymol groups, which were identified as *S. aureus* by 16S ribosomal DNA sequencing.

#### 2.7.4. Vancomycin in Combination with Thymol Restored the White Blood Cell (WBC) Counts in Mice

The White Blood Cell (WBC) counts in all groups, except in the PBS group, decreased, and then recovered to the normal range (0.8 × 10^9^/L–6.8 × 10^9^/L; Table 1). The WBC counts in the combination treatment groups were significantly (*p* < 0.05) different from those in the PBS-treated control group.

#### 2.7.5. Vancomycin in Combination with Thymol Reduced the Levels of Interleukin 6 (IL-6) and Tumor Necrosis Factor α (TNF-α)

**Figure 10:**
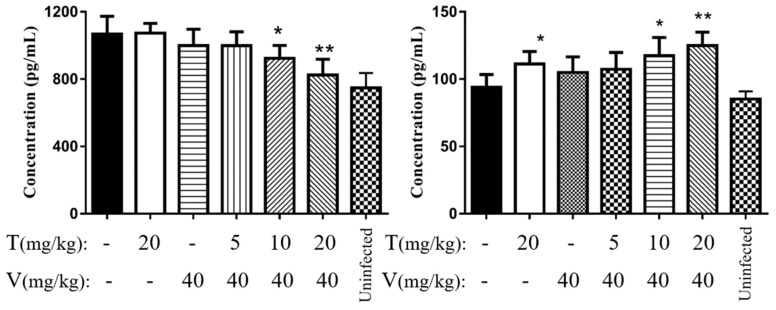
Serum THF-α and IL-6 levels in model mice after treatment. (**A**) THF-α; (**B**) IL-6. * *p* < 0.05, ** *p* < 0.01 versus the PBS (control) group. T, thymol; v, vancomycin hydrochloride.

The levels of the TNF-α and IL-6 are shown in Figure 10. Mice treated with a combination of vancomycin and thymol, especially in the high- and middle-dose groups, showed significant (*p* < 0.05) reduction in serum IL-6 and TNF-α levels. After 24 h of treatment, TNF-α levels were significantly (*p* < 0.05) decreased in the high- and middle-dose combination treatment groups, by 21.7% ± 14.8% (*p* < 0.01) and 17.2% ± 7.3% (*p* < 0.05), respectively, compared with that in the PBS group. IL-6 was significantly increased in the high- and middle-dose combination treatment groups by 26.5% ± 17.3% (*p* < 0.01) and 35.0% ± 24.1% (*p* < 0.05), respectively, compared with that in the PBS group.

## 3. Discussion

MRSA is a “superbug” that has spread globally. The first drug of choice for treating MRSA infections is vancomycin. However, with the emergence of vancomycin-resistant *S. aureus* (VRSA), MRSA has become an even more serious clinical issue [26]. In addition, MRSA can form biofilms, and biofilm bacteria are markedly less susceptible to antibiotics [27]. MRSA biofilms have become the greatest threat to critical care patients, especially patients in the intensive care unit (ICU), and patients with implanted medical devices or long-term wounds [28].

Compared to conventional antibiotics, thymol has a higher MIC, similar to that of emodin [29], baicalin [30], and other compounds extracted from plants. However, even at low concentrations, thymol has prominent anti-biofilm activity. Here, we used sub-MICs of thymol (8, 16, 32 and 64 μg/mL), which were determined based on the results of susceptibility testing. These concentrations showed no significant (*p* < 0.05) effect on the growth of MRSA, and thus applied less selective pressure. The median lethal dose (LD_50_) of thymol when administered by intraperitoneal injection is 608 mg/kg; therefore, these concentrations are safe for use in humans.

This study evaluated the effects of thymol upon the formation MRSA biofilms and the clearance of pre-existing biofilms. The results showed that thymol exhibited remarkable inhibitory effects against MRSA biofilms. To explore the underlying mechanism of action, we investigated the effects of thymol on PIA production, eDNA release, and biofilm-regulated gene expression. The life cycle of a biofilm has four stages: bacterial attachment, formation of microcolonies, maturation of the microcolonies into a biofilm, and cell dispersal [12,14,31]. PIA and eDNA are important components of MRSA biofilms [32]. PIA plays a crucial role in adhesion and aggregation [33]. It has been reported that although bacteria lacking PIA could adhere to biomaterials at the initial stage, at the later stage, they are unable to form a biofilm because cell-to-cell adhesion was greatly reduced. Similarly, eDNA is an essential component for biofilm formation, as it plays key roles in bacterial adhesion, aggregation, microcolony formation and biofilm architecture. The expression of PIA is regulated by the genes of the intercellular adhesive (*ica*) operon (*ica*ABCDR) and *sar*A [34]. The *ica*A gene, which encodes acetylglucosamine transferase [35], cannot be expressed in the absence of *sar*A, because sarA binds to the promoter of the *ica* operon [36,37,38]. Recent research has suggested that *cid*A and *sar*A also regulate biofilm formation and clearance through an *ica*-independent pathway, especially in MRSA [39,40,41,42]. Valle et al. [38] reported that the *sar*A mutation increased the expression of extracellular protease and nuclease, which reduced biofilm formation. Contrary to other findings, *agr* did not affect the biofilm-forming ability of *S. aureus* [38]. Therefore, we selected *ica*A, *cid*A and *sar*A for our study. We found that thymol reduced PIA synthesis and eDNA release, and RT-PCR showed that the transcript levels of these three genes were reduced in a dose-dependent manner after treatment with thymol. The changes in *sar*A, which regulates biofilm formation through various pathways, were the most obvious. These findings suggest that thymol is effective against TCH1516 biofilms.

Even after adopting various preventive measures, biofilm-associated infections have become commonplace. Once a biofilm is formed on a tissue or on the surface of an implanted medical device, it is nearly impossible to eliminate using conventional doses of antibiotics [43,44,45]. The situation is even more serious in the case of MRSA biofilm-associated infections. In such cases, combinatorial treatments are considered the first choice, as the dose of vancomycin, that is conventionally used to treat MRSA infections, should not be increased due to its toxicity. Thymol showed substantial inhibitory effects against biofilm formation and eliminated pre-existing biofilms. Therefore, clinical MRSA biofilm-associated infections might be cured with a combination of vancomycin and thymol. We extended our previous in vitro results by modeling a clinical MRSA biofilm-associated infection using an intraperitoneal foreign-body infection mouse model. We speculated that the biofilms on medical instruments may be damaged by thymol, and then vancomycin can penetrate the biofilms and kill the bacterial cells.

After treatment with either thymol or vancomycin alone, many bacteria were still adhered to the surface, and colony counting and SEM showed that the biofilms were not completely eliminated. However, after treatment with a combination of the two drugs, especially at a high dose of thymol, the destruction of the biofilm was obvious, and only a few bacteria still adhered. This result showed that thymol treatment alone could only eliminate some of the MRSA biofilm bacteria in vivo, and that thymol could improve the therapeutic effects of vancomycin on MRSA biofilm infections, in a combinatorial therapy.

Histopathological observation is a convenient way to diagnose disease and evaluate treatment efficacy. Thymol and vancomycin alleviated the pathological lesions associated with the biofilm-covered implants, which was consistent with the findings of other reports [46,47]. The results showed that mice in the high-dose combination treatment group had a good prognosis after treatment.

WBCs are an important indicator of inflammatory responses. In this study, both vancomycin and thymol reduced the WBC counts compared to those in the PBS group. Vancomycin might reduce inflammation by killing planktonic bacteria. However, it is unlikely that thymol reduced inflammation by killing bacteria because its MIC and MBC were too high for the administered doses to reach an effective concentration. Thus, thymol might reduce the inflammation via its anti-inflammatory activity.

TNF-α and IL-6 are widely used as markers to study inflammatory responses. TNF-α is one of the earliest and most important mediators of inflammation. IL-6 induces a humoral response, more specifically B cell differentiation and antibody production, as well as a cell-mediated response, more specifically T cell activation, proliferation and differentiation, and it participates in the immune response [48,49]. A previous study demonstrated that thymol inhibits TNF-α and IL-6 production via the nuclear factor-kappa B (NF-κB) signaling pathway in mice with an LPS-induced acute lung injury [46,50]. However, in this study, TNF-α levels were not significantly (*p* > 0.05) reduced in the thymol group, but were significantly (*p* < 0.05) decreased in the high- and middle-dose combination treatment groups. This might be due to a decrease in immune stimulation, as most of the biofilms were eliminated. Contrary to the findings of a previous report [46], the increase in IL-6 in the thymol group and the high- and middle-dose combination treatment groups might be due to the destruction of biofilms and the release of a higher number of bacteria, leading to the rapid cellular and humoral immune responses.

In summary, this study explored the ability of thymol to effectively inhibit and eliminate MRSA biofilms by reducing the synthesis of PIA and the release of eDNA in vitro. We confirmed that thymol could enhance the bactericidal activity of vancomycin by damaging biofilms. Thymol also improved the immune status of model mice, resulting in a good prognosis. As a monomeric drug with a well-defined chemical structure, thymol shows a potential for use as a novel drug against MRSA biofilm-associated infections. However, further studies on the pharmacokinetics, pharmacodynamics and toxicology of thymol must be performed before initiating any clinical trials. We will study more MRSA strains in the future to confirm whether these findings hold for all MRSA strains.

## 4. Materials and Methods

### 4.1. Bacterial Strain and Drug

The methicillin-resistant *Staphylococcus aureus* (MRSA) strain TCH1516 (ATCC BAA-1717) that is susceptible to vancomycin was obtained from the American Type Culture Collection. The strain was cultivated in brain heart infusion broth (BHI; Sigma, St. Louis, MO, USA). Thymol (>98% purity, HPLC; CAS No. 89-83-8) was purchased from Chengdu Herbpurify Co., Ltd. (Chengdu, China). A stock solution of thymol (40.96 mg/mL) was prepared in dimethyl sulfoxide (DMSO).

### 4.2. Ethics Statement

All animal studies were performed in accordance with the approved experimental practices and standards of the Animal Ethics Committee of Sichuan Agricultural University (Chengdu, China), and the experimental protocols were approved by (23 September 2016), and were conducted under the supervision of the Animal Care Committee (project code: 20160906).

Female 8-week-old Kunming mice (weighing 18–22 g) were obtained from Chengdu Dossy Experimental Animals Co., Ltd. (License No. SCXK [Sichuan] 2014-030). Before challenge, the animals were acclimated for 1 week under specific, pathogen-free (SPF) conditions.

### 4.3. Growth Curve and Checkerboard Assays

To generate a growth curve, overnight cultures of MRSA strain TCH1516 were inoculated into 500 mL of fresh BHI broth. When the OD_600_ of the cultures reached 0.3, the bacteria were sub-cultured, and thymol was added at final concentrations of 0, 8, 16, 32 and 64 μg/mL. These subcultures were incubated with continuous shaking (190 rpm), or under static conditions at 37 °C. At various time points, the OD_600_ of a 0.5 mL aliquot was measured on an ultraviolet–visible (UV-VIS) spectrophotometer (UNICO, Shanghai, China) [51].

A checkerboard assay was performed as previously described using TCH1516 treated with 2-fold dilutions of vancomycin hydrochloride (0–16 μg/mL) and thymol (0–256 μg/mL) [52,53]. The growth inhibitory effects of these compounds were evaluated based on FICI [54].

### 4.4. Biofilm Assessments

#### 4.4.1. Biofilm Inhibition Assay

Overnight cultures of TCH1516 were inoculated in BHI broth and grown to an OD_600_ of 0.6 in the presence of different concentrations of thymol (0, 8, 16, 32 and 64 μg/mL) with continuous shaking (200 rpm) at 37 °C. Then, a 10 μL aliquot of the bacterial culture and 290 μL of BHI broth containing 3% sucrose (BHI-S) and the 0, 8, 16, 32 and 64 μg/mL of thymol were mixed in a sterile, 96-well flat-bottom polystyrene microtitration plate (Solarbio, Beijing, China). After incubation for 24 h at 37 °C, non-adherent cells were removed, and a solution of 10% formaldehyde (100 μL) was added to cells and incubated overnight at 20 °C to fix the biofilms. After removing the formaldehyde, the wells were washed with phosphate-buffered saline (PBS) three times, and then 100 μL of 0.1% crystal violet (CV) was added to stain the biofilm. After incubation for 30 min at 20 °C, the plates were rinsed with ultrapure water and dried. Then, 200 μL of 33% acetic acid was added to each well, and the absorbance at 490 nm (A_490_) was determined on a microplate reader (Thermo Scientific, Waltham, MA, USA). The absorbance was used as a measure of the biofilm formation [55].

Scanning electron microscopy (SEM) was used to analyze the phenotypes and any morphological changes in the biofilms. The biofilms were cultured in 24-well plates containing glass coverslips using a previously described method [56]. After 24 h of culture, the biofilms on the coverslips were gently washed with sterile PBS and prepared for SEM. Finally, the biofilms were observed by SEM (Hitachi S4800N; Tokyo, Japan) [56].

#### 4.4.2. Biofilm Removal Assay

To evaluate the removal of pre-existing biofilms, 10 μL of bacterial solution (OD_600_ = 0.6) was mixed with 290 μL of fresh BHI-S in a sterile 96-well plate and incubated at 37 °C for 24 h to obtain mature TCH1516 biofilms. After removing the planktonic cells, the biofilms were washed with sterile PBS once, and then 300 μL of PBS containing different concentrations of thymol (0, 8, 16, 32 and 64 μg/mL) were added to each well. After 24 h of incubation at 37 °C, the same fixation, staining and biofilm measurement procedures were applied as described above (Section 4.4.1. Biofilm inhibition assay) were performed [57].

Bacterial solutions were incubated in 24-well plates containing glass coverslips at 37 °C for 24 h to allow mature biofilms to form on the coverslips. After gentle washing with sterile PBS, the coverslips were incubated with PBS containing different concentrations of thymol (0, 8, 16, 32 and 64 μg/mL) at 37 °C for 24 h. Then, the remaining biofilms on each coverslip were washed and prepared as described above.

To evaluate the biofilm clearance effect of the combinatorial treatment with vancomycin and thymol, mature biofilms were established using the method described above. After one wash with PBS, thymol and vancomycin hydrochloride were added to each biofilm-containing well. The final concentrations of vancomycin hydrochloride were 0, 0.25, 0.5, 1, 2 and 4 μg/mL, and the final concentrations of thymol were 0, 32, 64, 128, 256 and 512 μg/mL [58].

### 4.5. Polysaccharide Intercellular Adhesin (PIA) Assay

A 10 μL aliquot of bacterial solution (OD_600_ = 1.8) was inoculated on Congo red agar [59,60] containing different concentrations of thymol (0, 8, 16, 32, 64 and 128 μg/mL), and the plates were observed after incubation for 24 h at 37 °C [59].

### 4.6. Determination of eDNA Release

Bacterial culture (OD_600_ = 0.6) was added to a 96-well plate, and then treated with various concentrations of thymol (0, 8, 16, 32, 64, 128, 256 and 512 μg/mL). After incubation for 24 h at 37 °C, eDNA was extracted according to the method proposed by Rice [61]. Finally, eDNA release was evaluated by spectrophotometry with a micro-spectrophotometer (NanoDrop One, Thermo Scientific, Waltham, MA, USA).

### 4.7. Real-Time PCR

MRSA was cultured in BHI in the presence of different concentrations of thymol (0, 8, 16, 32, and 64 μg/mL) and grown to an OD_600_ = 1.8. Then, total RNA was extracted with an RNA kit (Tianmo Biotech, Beijing, China) according to the manufacturer’s instructions. Extracted RNA was quantified and tested for impurities by determining the absorbance at 260 nm (A_260_) and the A_260_/A_280_ ratio, respectively, on a Nanodrop spectrophotometer (Thermo). The extracted RNA was reverse transcribed using 5× All-In-One MasterMix (abm, Vancouver, BC, Canada) [62]. PCR was performed in a 20 μL reaction volume containing EvaGreen 2× qPCR MasterMix-No Dye (abm, Canada), according to the manufacturer’s instructions. Real-time PCR was performed on a CFX Connect™ Real-Time System (Bio-Rad Laboratories, Hercules, CA, USA) with specific primers (Table 2) designed using the Primers 5.0 software. The levels of the target transcripts were calculated relative to those of the 16S rRNA (housekeeping gene) by using the 2^−ΔΔ*C*t^ method [63].

### 4.8. Effects of Thymol on MRSA Infection in a Mouse Model of Intraperitoneal Foreign-Body Infection

#### 4.8.1. Implant Preparation

Implants (1 mm in length, 0.71 mm outer diameter, 0.41 mm inner diameter; Shifeng, China) were incubated with bacterial culture (OD_600_ = 0.05, MRSA in BHI-S) in a 50 mL conical flask for 24 h at 37 °C with shaking at 110 rpm to allow the bacteria adhere and form biofilms. Before implantation, the number of bacteria adhered to each implant was adjusted to an average of 2 × 10^4^ CFU/mL.

#### 4.8.2. Establishment of a Mouse Model

Intraperitoneal, foreign-body, biofilm infection models were established according to the procedure described by Christensen et al. [64]. For the surgical procedure, mice were anesthetized with pentobarbital (40 mg/kg) and shaved with a grainer. Then, the abdominal region was disinfected with polyvinyl pyrrolidone-I (PVP-I). A 0.6 cm incision was made in the left groin of the mouse to expose the abdominal cavity. The implants containing MRSA biofilms were washed with sterile PBS to remove planktonic cells. Then, the implants were placed into the abdominal cavity of the mice. Finally, the incision was sutured with a 4–0 silk thread. All wounds healed without any complications. After healing, the mice were randomly divided into six groups (*n* = 5 per group). In the thymol-treated group, the mice were administered thymol (20 mg/kg), and in the antibiotic monotherapy group, the mice were administered vancomycin hydrochloride (40 mg/kg). In all three combination treatment groups, the mice were administered vancomycin at 40 mg/kg, and thymol (injected) at doses of 5, 10 and 20 mg/kg in the low-, middle- and high-dose groups, respectively. In the control group, the mice were administered isochoric sterile PBS. 30 min after the operation, we started to inject different drugs and PBS. All drugs and PBS were intraperitoneally injected twice a day. The volume of all injected drugs was 0.1 mL.

#### 4.8.3. White Blood Cell (WBC) Counts

24 h after the first treatment, blood samples were collected by retro-orbital bleeding, and WBCs were counted using a blood cell analyzer (BC-2800Vet; Mindray, Shenzhen, China) in accordance with the manufacturer’s instructions. Then, all the mice were euthanized.

#### 4.8.4. Colony Counting and SEM

To evaluate the biofilm formation in implants in response to various treatments, the implants were removed from the peritoneal cavity, and the bacteria present on the implants were calculated by colony counting, in accordance with the method proposed by Luo [65]. Then, the implants were prepared for SEM [66].

#### 4.8.5. Histopathological Observations

For the histopathological studies, the peritoneal tissues surrounding the implants in all model mice were carefully separated and fixed in 10% formalin (Solarbio, Beijing, China). After processing, the tissues were sectioned with a microtome and stained with hematoxylin and eosin (H&E) for microscopic observation [67].

### 4.9. ELISA

An enzyme-linked immunosorbent assay (ELISA) was performed to determine the levels of IL-6 and TNF-α in serum according to the manufacturer’s instructions (Lianke, Hangzhou, China).

### 4.10. Statistics

All experiments, except the animal assays, were repeated at least three times, and the average value of all experiments was used. All data are presented as the mean ± standard deviation (SD), and the statistical significance of differences was analyzed by an unpaired two-tailed Student’s *t*-test or deviation analysis, and analysis of variance (ANOVA) using GraphPad Prism 7 software. Differences with *p* values less than 0.05 and 0.01 were considered statistically significant and extremely significant, respectively.

### 4.11. Data Availability

All data generated and/or analyzed in the current study are included in this article or in the supplementary information files, or are available from the corresponding author upon reasonable request.

## Figures and Tables

**Table 1:** White Blood Cell (WBC) counts in mice in response to different treatments.

Group	Treatment	WBC Counts (10^9^/L)
A	PBS	7.42 ± 1.94
B	Thymol (20 mg/kg)	5.10 ± 0.98 *
C	Vancomycin hydrochloride (40 mg/kg)	6.72 ± 1.67
D	Vancomycin hydrochloride (40 mg/kg) + thymol (5 mg/kg)	4.08 ± 1.89 *
E	Vancomycin hydrochloride (40 mg/kg) + thymol (10 mg/kg)	3.24 ± 1.02 **
F	Vancomycin hydrochloride (40 mg/kg) + thymol (20 mg/kg)	4.30 ± 2.27 *
G	Uninfected	5.52 ± 1.43

Results are expressed as the mean ± standard deviation. Asterisks indicate a significant difference compared with the PBS group (* *p* < 0.05 and ** *p* < 0.01).

**Table 2:** Real-time polymerase chain reaction (RT-PCR) primers.

Primer	Sequence (5′→3′)	Product/bp
icaA-F	TTTCGGGTGTCTTCACTCTAT	229
icaA-R	CGTAGTAATACTTCGTGTCCC	
cidA-F	GATTTTTCATCTTCCCTTAGCCG	300
cidA-R	GCGTCTACACCTTTACGATGTTTAT	
sarA-F	TTGTTTTCGCTGATGTAT	100
sarA-R	CAATGGTCACTTATGCTG	

## Supplementary Materials

Supplementary materials can be found at https://www.mdpi.com/2076-2607/8/1/99/s1.

## Abbreviations

MRSA	methicillin-resistant *Staphylococcus aureus*
PIA	polysaccharide intracellular adhesion
eDNA	extracellular DNA
SEM	scanning electron microscope
WBC	white blood cell
IL-6	interleukin 6
TNF-α	tumor necrosis factor α
OD	optical density
FICI	fractional inhibitory concentration index
BHI	brain heart infusion
LD_50_	median lethal dose
