# Supplementary material for: Thymol Inhibits Biofilm Formation, Eliminates Pre-Existing Biofilms, and Enhances Clearance of Methicillin-Resistant Staphylococcus aureus (MRSA) in a Mouse Peritoneal Implant Infection Model
Source: Microorganisms. 2020 Jan 10;8(1):99. doi: 10.3390/microorganisms8010099 (PMC7023310; doi:10.3390/microorganisms8010099)
Supplement: Supplementary file 1 [file microorganisms-08-00099-s001.zip › suppl/table S1.docx]

Table S1. Checkerboard assays showing the additive effect of thymol on the vancomycin hydrochloride MIC against MRSA strain TCH1516.

|  | 1 | 2 | 3 | 4 | 5 | 6 |
| --- | --- | --- | --- | --- | --- | --- |
| A | T_256_  V_2_ | T_128_  V_2_ | T_64_  V_2_ | T_32_  V_2_ | T_16_  V_2_ | T_0_  V_2_ |
| B | T_256_  V_1_ | T_128_  V_1_ | T_64_  V_1_ | T_32_  V_1_ | T_16_  V_1_ | T_0_  V_1_ |
| C | T_256_  V_0.5_ | T_128_  V_0.5_ | T_64_  V_0.5_ | T_32_  V_0.5_ | T_16_  V_0.5_ | T_0_  V_0.5_ |
| D | T_256_  V_0.25_ | T_128_  V_0.25_ | T_64_  V_0.25_ | T_32_  V_0.25_ | T_16_  V_0.25_ | T_0_  V_0.25_ |
| E | T_256_  V_0.125_ | T_128_  V_0.125_ | T_64_  V_0.125_ | T_32_  V_0.125_ | T_16_  V_0.125_ | T_0_  V_0.125_ |
| F | T_256_  V_0_ | T_128_  V_0_ | T_64_  V_0_ | T_32_  V_0_ | T_16_  V_0_ | T_0_  V_0_ |

Different combinations of doubling concentrations of thymol (T), from 0 to 256 µg/ml, and vancomycin hydrochloride (V), from 0 to 2 µg/ml, were tested. No bacterial growth is indicated in the table as a yellow box.
