# Supplementary material for: Thymol Inhibits Biofilm Formation, Eliminates Pre-Existing Biofilms, and Enhances Clearance of Methicillin-Resistant Staphylococcus aureus (MRSA) in a Mouse Peritoneal Implant Infection Model
Source: Microorganisms. 2020 Jan 10;8(1):99. doi: 10.3390/microorganisms8010099 (PMC7023310; doi:10.3390/microorganisms8010099)
Supplement: Supplementary file 1 [file microorganisms-08-00099-s001.zip › suppl/table S2.docx]

Table S2. The removal effect of vancomycin and thymol on pre-existing biofilms.

| *c*(Vancomycin hydrochloride)/(μg/mL) | *c* (thymol)/(μg/mL) | | | | | | | |
| --- | --- | --- | --- | --- | --- | --- | --- | --- |
|  | 0 | 32 | 64 | 128 | 256 | | | 512 |
| 0 | 1.530±0.334 | 1.069±0.155 | 0.946±0.191 | 0.838±0.132 | | 0.689±0.039 | | 0.461±0.028 |
| 0.25 | 1.630±0.370 | 1.200±0.185 | 1.193±0.170 | 1.122±0.277 | | 0.919±0.058 | 0.426±0.082 | |
| 0.50 | 1.444±0.215 | 1.098±0.093 | 1.245±0.176 | 1.216±0.405 | | 0.847±0.029 | 0.430±0.010 | |
| 1.00 | 1.139±0.168 | 1.005±0.077 | 0.790±0.024 | 0.648±0.054 | | 0.567±0.109 | 0.417±0.029 | |
| 2.00 | 0.988±0.135 | 0.806±0.159 | 0.672±0.080 | 0.612±0.104 | | 0.462±0.027 | 0.414±0.010 | |
| 4.00 | 0.561±0.135 | 0.546±0.159 | 0.519±0.080 | 0.492±0.104 | | 0.452±0.027 | 0.426±0.010 | |
