# Supplementary figures and images for: Thymol Inhibits Biofilm Formation, Eliminates Pre-Existing Biofilms, and Enhances Clearance of Methicillin-Resistant Staphylococcus aureus (MRSA) in a Mouse Peritoneal Implant Infection Model
Source: Microorganisms. 2020 Jan 10;8(1):99. doi: 10.3390/microorganisms8010099 (PMC7023310; doi:10.3390/microorganisms8010099)

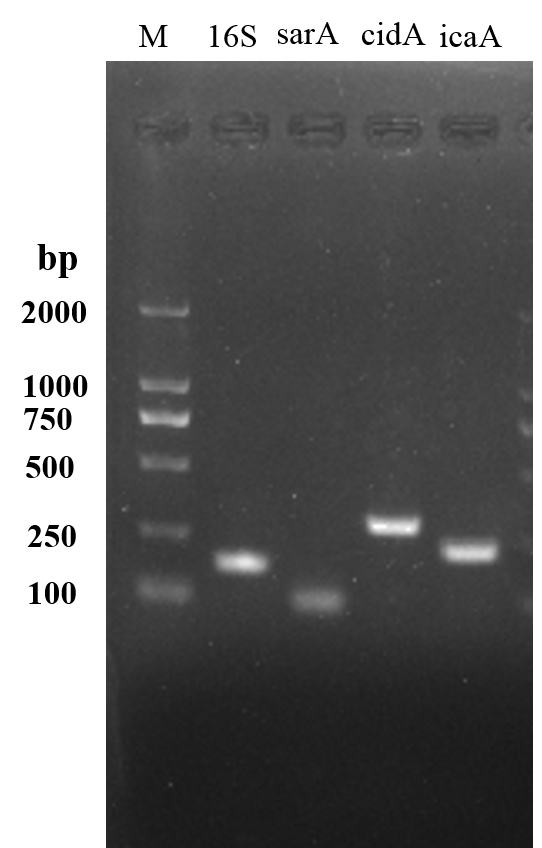

Supplement: Supplementary file 1 [file microorganisms-08-00099-s001.zip › suppl/Figure S1.tif]
